# Self-help Digital Interventions Targeted at Improving Psychological Well-being in Young People With Perceived or Clinically Diagnosed Reduced Well-being: Systematic Review

**DOI:** 10.2196/25716

**Published:** 2022-08-26

**Authors:** Camilla M Babbage, Georgina M Jackson, E Bethan Davies, Elena Nixon

**Affiliations:** 1 National Institute for Health and Care Research (NIHR) MindTech Medtech Co-operative Mental Health & Clinical Neurosciences Institute of Mental Health, University of Nottingham Nottingham United Kingdom; 2 Institute of Mental Health, Mental Health & Clinical Neurosciences University of Nottingham Nottingham United Kingdom

**Keywords:** digital health interventions, psychological well-being, mental well-being, mental disorders, mental health, children and young people, self-help, systematic review

## Abstract

**Background:**

Levels of well-being are declining, whereas rates of mental health problems remain high in young people. The World Health Organization defines mental health as not merely the absence of mental disorder but also includes social and psychological well-being as integral to positive mental health, highlighting that mental health is applicable to young people with mental health conditions *and* those without a diagnosis of a mental health condition. Reduced mental well-being have been identified in studies of young people with clinical populations, as well as in populations consisting of nonclinical young people. Self-help digital interventions can be delivered at mass at a low cost and without the need for trained input, thereby facilitating access to support for well-being. Self-help interventions are effective in young people with mental health conditions, but systematic reviews of such studies have been limited to randomized controlled trials, have not included reduced well-being as an inclusion criterion, and do not consider engagement factors such as retention.

**Objective:**

The objective of this study was to systematically review all controlled studies of digitally delivered, self-administered interventions for young people aged 9 to 25 years, with perceived or clinically diagnosed reduced psychological well-being. Participant retention and effectiveness of the interventions were also explored.

**Methods:**

A systematic search of the PsycInfo, EMBASE, Cochrane, Scopus, and MEDLINE databases from inception to 2021, reference searches of relevant papers, and gray literature was carried out for digitally controlled studies conducted with young people with perceived or clinically diagnosed reduced well-being, aimed at improving psychological well-being. Data were extracted to identify the effectiveness and retention rates of the interventions and the quality of the studies.

**Results:**

Overall, 1.04% (12/1153) of studies met the inclusion criteria: 83% (10/12) of studies were randomized controlled trials and 17% (2/12) were controlled pre-post studies. Most (6/12, 50%) studies aimed to improve symptoms of depression; 3 interventions aimed at both anxiety and depressive symptoms and 2 studies aimed at improving social functioning difficulties. Owing to the high risk of bias across interventions and lack of similar outcome measures, a meta-analysis was not conducted. Retention rates across studies were regarded as good, with moderate to high retention. Overall, the findings indicated that predominantly self-administered self-help interventions improved well-being in the areas targeted by the intervention and identified additional areas of well-being that were positively affected by interventions. Few interventions supported psychological well-being that was different from those used by young people with a clinical diagnosis of mental illness or young people from neurodiverse backgrounds.

**Conclusions:**

The findings, along with the advantages of self-help interventions, highlight the need for upscaling self-help interventions to better support vulnerable populations of young people who experience poor psychological well-being.

**Trial Registration:**

PROSPERO CRD42019129321; https://tinyurl.com/4fb2t4fz

## Introduction

### Background

Well-being is a multifaceted construct consisting of a person’s physical, psychological, cognitive, social, and economic well-being [1,2]. Psychological and social well-being (termed hereon as psychological well-being) are identified as being integral to a person’s health by the World Health Organization, which highlights that mental health does not merely require the absence of a mental health condition but also extends to a need to experience positive well-being, which is inclusive of those not living with a mental health condition. Poor well-being has been identified in both populations of young people living with mental health conditions and those who are not [3-6]. Indicators of psychological well-being and mental health have been found to be similar in youth populations, for example, relationships with teachers, supportive families, and engaging with school are related to good psychological well-being and mental health, and bullying, single parent families, peer problems, and parent-child arguments are indicators of poor psychological well-being and mental illness [1,2,7]. Those living with a mental health condition are more likely to experience reduced well-being compared with those not living with a mental disorder, but there is alarming evidence to suggest that nonclinical youth populations also show trends toward reduced well-being [4,8]. In light of the rising decline in psychological well-being and increasing levels of mental illness in young people [9,10], the efficacy and suitability of well-being interventions that are already available for those with mental health conditions should also be considered in young people who experience low psychological well-being without mental health conditions [11].

The calls for increased resources for young people with reduced well-being may be at odds with the current state of the health care system. Young people aged 10 to 24 years have the lowest health care coverage compared with other age groups [12], and service providers tend to face significant barriers to delivering support, including issues stemming from a lack of investment, resources, and training, resulting in decreased service-user access and increased waiting times for child mental health services [13,14]. The use of self-help interventions is recommended by health care professionals for young people with depression according to the national guidelines in the United Kingdom [15], and increased recognition of self-help is leading to its greater implementation within mental health care [16]. This follows the stepped-care approach which proposes that an individual should initially be treated with the least intensive and least expensive intervention; for example, self-help, which can increase at a rate proportional to the individual’s need [17]. Considering the dearth of resources available for psychiatry, resources available for well-being would likely be even fewer [18]. Thus, self-help interventions would be appropriate for young people experiencing reduced well-being. In addition, the introduction of digital health interventions (DHIs) could overcome the current challenges experienced by health care systems worldwide by improving access to care and promoting healthier populations [14], as digitally delivered self-help enables large-scale delivery at a low cost [19]. Furthermore, DHIs help overcome other barriers experienced by young service users in accessing care services, including stigma and embarrassment [20]. Digital self-help for adolescents who prefer to be self-reliant may give them feelings of autonomy [21,22], and concerns about stigma or embarrassment could be reduced, as DHIs can be used privately and anonymously [20,23,24]. The provision of DHI support is consistent with international guidelines that recognize the value of DHI in advancing universal health care coverage [14] and supports the call for early well-being interventions for young people with mild or low-level needs who would benefit from support [25].

The recent surge in self-help DHI has resulted in an increasing number of reviews of studies assessing their effectiveness [26]. Most of these reviews have examined guided self-help interventions, which entail a degree of web-based or offline guidance from a therapist rather than purely self-help interventions. Self-help interventions, by strict definition, are delivered without the need for any professional support through text, audio, video, group, or individual exercises [27]. Newman et al [28] categorized the degree of therapist involvement into 3 levels of support. The lowest level included completely self-guided interventions, with the second level additionally including the therapist providing rationale and instructions for using the intervention. The third level of involvement entailed the greatest amount of support, with the therapist actively involved in providing therapeutic support during the intervention. Considering the level and type of support provided by professionals is important when trying to balance the accessibility, adherence, and efficacy of therapy [29]. Digital self-help interventions vary in their content, usually adapting psychological therapy into a digital format, such as computerized cognitive behavioral therapy (CBT) [30], and also include other types of interventions, such as bibliotherapy [31], serious games [32], peer-to-peer support [33], self-monitoring, and medication adherence [34], all aimed at improving well-being in young people. Current systematic reviews have been limited in terms of their scope and coverage of the level of self-help support involved, with reviews tending to focus on therapist-guided self-help interventions in young people and in young people with clinically diagnosed depression and anxiety [35-39].

The only published systematic reviews to explore the efficacy of therapist support in self-help intervention studies were by Bennett et al [16] and Grist et al [36]. The study by Bennett et al [16] reviewed both paper-based (eg, bibliotherapy) and digital self-help interventions, whereas the study by Grist et al [36] identified digitally delivered interventions only, for young people with diagnosed mental health conditions. Both reviews found improved outcomes in randomized controlled trials (RCTs) with increased levels of therapist support; however, we do not know if this would also apply to young people who may experience reduced psychological well-being without a clinical diagnosis of mental illness. With concerns about the impact of the COVID-19 pandemic on young people, the use of digital technology to support young people’s well-being has been promoted [40,41]. Therefore, there could be an increase in the number of interventions available for young people experiencing reduced well-being, causing these reviews to become outdated. Considering the narrower focus on digital interventions fitting the 2 lower levels of support for self-guided interventions, as proposed by Newman et al [28], increasing the search criteria to potentially influential controlled before and after studies and to all common mental disorders in young people, could also be useful in identifying further relevant trials.

### Objectives

The objective of this systematic review was to explore the efficacy of predominantly self-administered digital interventions for young people experiencing perceived reduced well-being, whether or not they are diagnosed with a common mental disorder. Through the focus on young people with perceived reduced well-being, not excluding controlled pre-post studies and the inclusion of only digital self-administered interventions, this review will expand the scope of both studies by Bennett et al [16] and Grist et al [36]. The level of retention of self-help interventions will also be reported, which has not been explored in previous studies. If effective, such interventions would not rely on professional support or clinical diagnoses, expanding their scope and enhancing access to well-being support for vulnerable young people with reduced well-being.

## Methods

### Overview

Studies that assessed the effectiveness and acceptability of self-help digital interventions targeting young people with reduced well-being, with or without a diagnosis of a mental disorder, were included in the search.

A protocol was created and registered on PROSPERO, the International Register of Systematic Reviews (ID: CRD42019129321). Initially, the search focused on the neurodevelopmental conditions of tic disorders and associated conditions. However, because of the lack of self-help digital intervention studies for this condition and the relevance of the literature on all common mental health conditions in youth, the protocol was revised to apply the search criteria to all common youth mental disorders, as well as to perceived poor psychological well-being in the absence of a clinical diagnosis.

### Types of Studies

The search was limited to publications in English because translation was not possible. The search was intended for quantitative studies; other study types, including qualitative studies, reviews, commentaries, theses, and protocols, were excluded. The study designs were required to be controlled pre-post intervention designs to be included, but randomization or control groups were not mandatory. Studies using secondary data, in which the primary paper had already been included, were removed to prevent bias.

### Types of Participants

#### Age

Studies were included if the sample was aged between 9 and 25 years and had a mean population age of ≥18.51 years. This ensured that all interventions were performed by young people [16].

#### Conditions

To be included, the sample of young people had to be identified by the authors of the study or identified as having perceived reduced well-being through self-selection. Authors of the study may have used a threshold for a well-being measure, symptom severity or rating scales for mental conditions, or diagnosis of a mental disorder indicative of reduced well-being. Studies in which well-being was not measured (ie, prevention studies) were excluded. Data from subgroups that met the aforementioned criteria were included. Patients with physical health conditions (eg, epilepsy, pain management, and asthma) were excluded.

### Types of Interventions

The interventions had to meet the criterion of being a digital self-help intervention for well-being. This included both web-based and offline digital interventions.

Studies adopting a combined design of digital and nondigital interventions were excluded. No exclusion criteria were applied for the type of digital delivery, length, or number of intervention sessions.

To meet the definition of self-help, interventions should require minimal support from a therapist [27]. Using the categories proposed in the study by Newman et al [28], interventions should fit the 2 lower levels of support and be completely or predominantly self-administered. Therefore, interventions with greater than predominantly self-administered support (ie, therapeutic support from a trained professional) were excluded. Interventions with support from gatekeepers or research staff for technical assistance or overseeing the practical provision of the intervention were included, as this did not provide therapeutic support. Studies including automated emails or feedback were included; however, responses containing therapeutic feedback or guidance were excluded. Many studies on young people have included safety checks. As safety checks do not offer therapeutic support and are necessary for ethical conduct, studies were not excluded based on their use.

The aim of the intervention required improving the psychological well-being of young people, identified by an outcome measure that pertained to a respective measure of well-being.

### Types of Outcome Measures

The primary outcome of interest was psychological well-being. Studies that did not measure changes in well-being (ie, pre-post intervention) were excluded, as this would not allow the assessment of the effectiveness of the intervention.

The outcome measures used in each study were documented to explore how psychological well-being was measured. In defining *psychological well-being* this study used the 2 domains of *psychological* and *social* well-being, as they are areas of functioning greatly affected when young people have a mental health condition which presents with psychological symptoms. The study by Pollard and Lee [42] documented indicators of psychosocial domains of well-being, including aggression, anxiety, emotional problems, loneliness, psychological distress, coping, fulfillment, happiness, purpose in life, and self-esteem. Indicators of social well-being included antisocial behavior, negative life events, peer problems, troubled home relationships, prosocial behaviors, quality of life, relationships with peers, social skills, and support. For a full list of indicators, refer to the study by Pollard and Lee [42]. The secondary outcomes of the effectiveness and trial retention were also recorded.

### Search Methods

#### Overview

The search strategy covered the following constructs: (1) young people, (2) mental health conditions, (3) modes of digital intervention and delivery, and (4) self-help (Multimedia Appendix 1). These constructs were searched using the “AND” Boolean term, meaning papers had to meet each of the above constructs to be returned by the database. Each concept included wide-reaching terms, aiming to capture as much literature as possible. Furthermore, terms were adapted depending on the relevant subject headings or Medical Subject Headings terms to include plurals, singular words, and different spellings of the words.

#### Electronic Searches

Five bibliographic databases (PsycInfo, Embase, Cochrane, Scopus, and MEDLINE) were electronically searched for relevant articles from the conception of the database until September 24, 2021, when the search was conducted. An example of the search strategy is presented in Multimedia Appendix 2.

#### Other Searches

Reference lists of relevant systematic reviews (Multimedia Appendix 3 [16,21,31,32,34,35,37,39,43-45]) were hand searched by CMB and imported into EndNote (Clarivate) with other references. The OpenGray database was searched to identify any relevant gray literature.

### Data Synthesis

#### Selection of Studies

**Figure 1:**
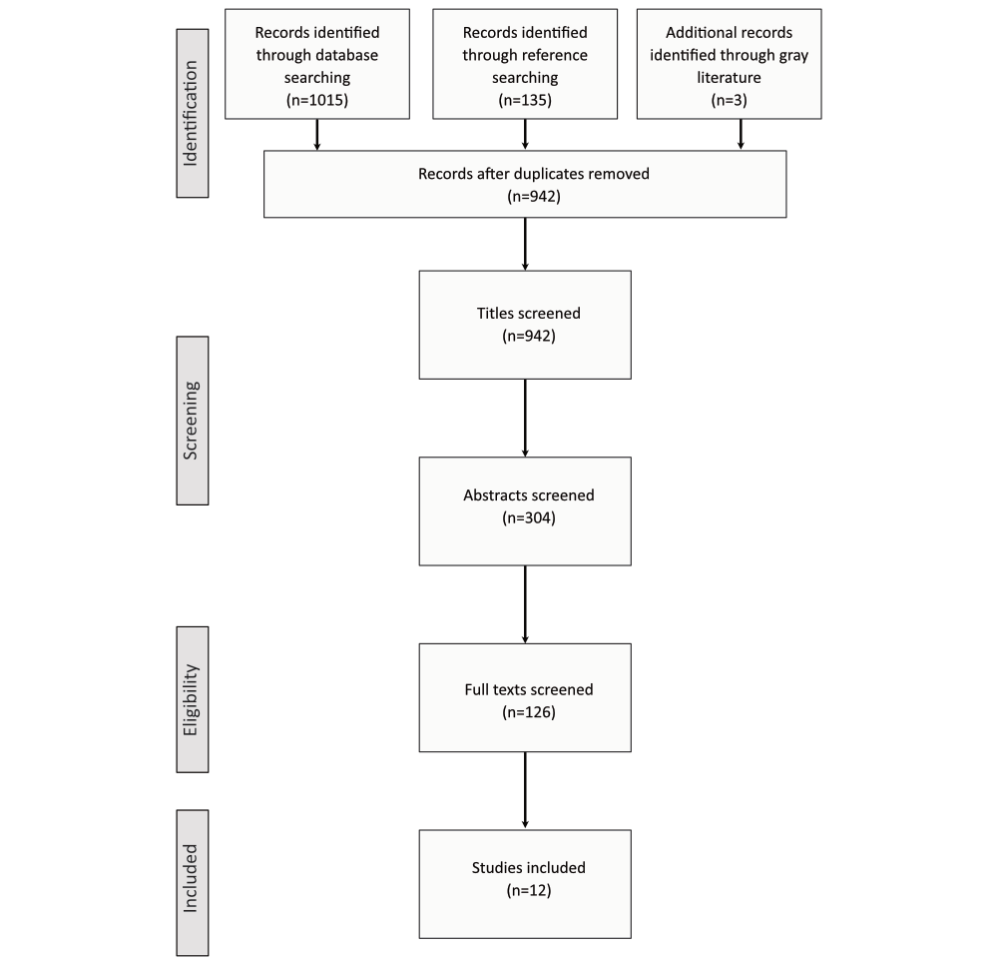
Flowchart of studies in line with PRISMA (Preferred Reporting Items for Systematic Reviews and Meta-Analyses) guidelines.

All references were imported into EndNote X9, and duplicate references were removed. CMB and EBD screened the references based on the title, excluding references that did not fit the inclusion criteria and documenting reasoning in the flowchart shown in Figure 1. This process was repeated based on abstracts and full papers. During full-paper screening, all remaining articles were reviewed by both CMB and EBD. The authors were contacted when more information was required to indicate inclusion or exclusion. Disagreements were resolved through discussions between CMB and EBD.

Full-text articles were screened according to eligibility criteria. Overall, 20.6% (26/126) of the relevant full-text articles were independently screened by the authors GMJ and EN to check for agreement. This systematic review used the PRISMA (Preferred Reporting Items for Systematic Reviews and Meta-Analyses) statement to ensure transparent and comprehensive reporting [46].

#### Data Extraction and Management

Data extraction of papers meeting the inclusion criteria was extracted into RevMan (version 5.3) by CMB. Data extracted included sample characteristics (age range, total number, gender, and inclusion criteria), trial retention (by noting the number of participants retained based on those completing outcome measures after the intervention and at follow-up and weighting this by sample size) and intervention retention (ie, the percentage of participants who completed the intervention), study design, intervention and comparison intervention details (aim, length, number of sessions, components of intervention, professional contact, and delivery method), outcomes measured, and when these were measured; key findings (including means, SDs, CIs, and *P* values) were taken from each study and placed into a summary of findings table.

#### Assessment of Risk of Bias

The risk of bias for each study was assessed by CMB using the Cochrane Collaboration tool [47], which covers 6 domains of bias including selection bias, performance bias, detection bias, attrition bias, reporting bias, and other biases. Disagreements were discussed with EN and GMJ and resolved by consensus. Experimenter bias was assessed as a source of bias. This tool was developed for RCT studies, but for this review, the tool was also used in controlled pre-post studies, with adaptations to the tool suggested in previous protocols [48]. CMB made judgments on the risk of bias, including a supporting quote from the text when possible, and if unknown, it should be stated. The attribution of low risk means that the bias was unlikely to have caused an effect on the findings, and high risk suggests that bias may have had a significant effect on the findings. No publications were excluded based on quality because a meta-analysis was not performed on the data. Studies with high bias were given less weighting when applying the findings to narrative synthesis.

## Results

### Study Selection

In total, 1153 studies were returned from the search, of which 63 (5.46%) were duplicates and were removed. Subsequently, of 1153 studies, 942 (81.7%) were screened by title: 618 (53.6%) were excluded based on title, 178 (15.44%) were excluded based on abstract, and 114 (9.89%) were excluded based on screening of full texts, leaving 12 (1.04%) papers included in the review. The reason for exclusion at each stage was documented, and the flow diagram is shown in Figure 1.

### Study Characteristics

Table 1 summarizes the study designs for all studies. In all, 10 studies were RCTs and 2 were controlled pre-post intervention studies, all published between 2006 and 2020. The sample size of young people with reduced well-being at baseline ranged from 23 to 240 (mean 107). Studies were categorized according to the primary aim of the interventions, which included improving depression, depression and anxiety, and social functioning. Half of the studies (6/12, 50%) aimed to improve symptoms of depression in young people experiencing depression and used 3 different interventions: *SPARX* [49-51], *The Journey* [52], and *MoodGYM* [53,54]. Three interventions, *Mobiletype*, *Stressbusters*, and *Shamiri-Digital* were aimed at both anxiety and depression; *Shamiri-Digital* and *Mobiletype* were explored in one study [55,56]. *Mobiletype* aimed to improve levels of anxiety, depression, and stress in young people showing elevated depression [56], and Shamiri-Digital was a universal intervention aimed at improving anxiety and depression [55]. *Stressbusters* aimed to improve anxiety and depression in young people experiencing low mood and depression, as assessed in 2 studies [57,58]. The final 2 studies explored social functioning difficulties with an intervention on blogging about social difficulties for a group of young people scoring below average on peer relationships [59] and a cognitive bias modification training (CBMT) intervention for participants scoring above the cutoff for social phobia [60]. The studies were conducted in Israel (n=1), New Zealand (n=3), Australia (n=2), the Netherlands (n=2), Norway (n=1), Kenya (n=1), and the United Kingdom (n=2).

**Table 1:** Summary of included studies, including sample age, study design, outcomes and key findings, and average age calculated as mean weighted by sample size.

Aim and authors; intervention name			Sample					Intervention components; control components			Delivery method; design		Key findings	
			Participants (males); age range (mean)		Inclusion criteria									
**Depression**														
	Merry et al [50]; SPARX^a^	N=187 (64); 12-19 (15.6)		>30 on CDRS-R^b^		CBT^c^, psychoeducation, relaxation skills, problem-solving, activity scheduling, challenging and replacing negative thinking, and social skills (n=104); treatment as usual (n=83)			PC; RCT^d^					SPARX group showed improvements on the CDRS-R (Cohen d=−0.293; *P*=.08) and significant improvements on MFQ^e^ [61] (*P*=.03), hopelessness (K-10; *P*=.04), and anxiety (SCAS^f^ 0.075); maintained at follow-up SPARX would be recommended and felt it had appeal
	Fleming et al [49]; SPARX	N=32 (18); 13-16 (14.9)		>30 on CDRS-R		CBT, psychoeducation, relaxation skills, problem-solving, activity scheduling, challenging and replacing negative thinking, and social skills (n=20); WL^g^ (n=12)			PC; RCT					SPARX showed significant improvements for depression (CDRS-R: F=18.11; *P*<.001; RADS^h^: F=4.13; *P*=.05) SPARX may be effective in treating students in special education services with minimal symptoms of depression and anxiety for depression No differences found at follow-up
	Poppelaars et al [51]; SPARX	N=208 (0); 11-16 (13.4)		>59 on RCADS^i^		CBT, psychoeducation, relaxation skills, problem-solving, activity scheduling, challenging and replacing negative thinking, and social skills; CBT program (n=50); SPARX+CBT program (n=56); MC^j^ (n=51)			PC; RCT					Significant reductions in RADS across groups with a medium effect to the 1-year follow-up No significant differences between groups for SPARX (Cohen d=−0.283; *P*=.11), CBT, or control
	Stasiak et al [52]; The Journey	N=34 (20); 13-18 (15.2)		>30 on CDRS-R or >76 on RADS-2		CBT, linking thoughts and actions to feelings, behavioral activation, pleasant activity scheduling, problem-solving and conflict resolution, cognitive restructuring, challenging unhelpful thoughts, thought stopping, relaxation techniques and, relapse prevention (n=17); AC^k^ (n=17)			PC; RCT					Significant improvement for The Journey depression ratings (CDRS-R: Cohen d=−0.532; *P*=.001) and problem-solving (Adolescent Coping Scale-Short [62]) Nonsignificant reductions found in the RADS for depression and quality of life (Pediatric Quality of Life Enjoyment and Satisfaction Questionnaire [63]) Suggests short-term efficacy and good adherence Secondary measures rarely met significance
	O'Kearney et al [54]; MoodGYM	N=23/78^l^ (23); 15-16 (—^m^)		>16 on the CES-D^n^		CBT, Information, relaxation, problem-solving, dysfunctional thoughts, negative thinking, assesses self-esteem, cognitive restructuring, assertiveness, and coping with relationships (n=23); AC (n=24)			Web; pre-post					Reduction of depression vulnerability after treatment for participants at risk compared with control group (CES-D: Cohen d=0.042; *P*=.87 and The Revised Children’s Attributional Style Questionnaire) Reductions maintained at follow-up compared with before the intervention Small sample size, so caution is noted
	Lillevoll et al [53]; MoodGYM	N=198/1337 (—); 15-20 (16.8)^l^		>16 on the CES-D		CBT, Information, relaxation, problem-solving, dysfunctional thoughts, negative thinking, assesses self-esteem, cognitive restructuring, assertiveness, and coping with relationships; MoodGYM without reminders (n=176); MoodGYM with reminders (n=176); MoodGYM with tailored reminders (n=175); WL (n=180)			Web; RCT					Participants with elevated depression (CES-D) did not show increased self-esteem (Norwegian version of the General Self-Efficacy Scale [64]) or reduced risk of depression (CES-D: *P*=.36) High attrition and adherence problems
**Anxiety and depression**														
	Reid et al [56]; Mobiletype	N=118 (32); 14-24 (18.1)		Mild or severe mental health indicated by a general practitioner or >16 on The Kazdin hopelessness scale for children [65].		Prompted to complete an entry on current activity; a beep emitted from the phone at random intervals with a reminder beep 5 minutes later; stress and mood 4 times a day; alcohol, drug use, sleep, diet, and exercise once a day (n=69); AC (n=49)			Mobile; RCT					The intervention group showed a main effect of time on the Emotional Self-Awareness Scale compared with attentional control. A significant effect of time was found at 6-week follow-up. The sample as a whole showed a decrease in mood-related scores for the Depression Anxiety Stress Scale [66], which may have been because of 91% of the sample receiving a pathways to care before the intervention in their pretest medical review.
	Wright et al [57]; Stressbusters	N=91 (31); 12-18 (15.4)		>20 on the MFQ		Goal-setting, getting activated, emotional recognition, noticing thoughts, thought challenging, problem-solving, improving social skills, and relapse prevention (n=46); AC (n=45)			PC; RCT					Nonsignificant MFQ reduction scores for Stressbusters (Cohen d=−0.172; nonsignificant), which plateaued after the first 4 sessions Nonsignificant increase in MFQ seen in the control group No significant differences at the 4-month follow-up (Beck Depression Inventory, SCAS, or MFQ)
	Smith et al [58]; Stressbusters	N=112 (—); 12-16 (—)		>20 on the MFQ-C		Goal-setting, getting activated, emotional recognition, noticing thoughts, thought challenging, problem-solving, improving social skills, and relapse prevention (n=55) WL (n=57)			PC; RCT					Stressbusters showed significant decrease in MFQ (Cohen d=0.097; *P*=.001) and Screening for Child Anxiety Related Emotional Disorders [67] for self-rated and parent-rated scores, compared with the control Attainment was significantly improved for Stressbusters
	Osborn et al [55]; Shamiri-Digital	N=56/103; N=49/103; 13-18 (—)		>10 on the Patient Health Questionnaire-8 [68]; >10 on Generalized Anxiety Disorder-7		Growth mindset, gratitude, and value or virtue affirmation (n=28; n=24); AC (n=28; n=25)			PC; RCT					A significant time×condition effect was found, suggesting greater reductions in those with depressive symptoms from baseline to follow-up (2 weeks) than those in the control group. A significant time effect was found for those with elevated anxiety; showed declines, regardless of group For both the elevated depression and anxiety group, changes surpassed the reliable-change index suggesting they met the standard for clinically reliable change
**Social functioning**														
	Boniel-Nissim and Barak [59]; Blogging	N=161 (37); 14-17 (15.5)		Scored lower on SD of index of peer relationship; interested in starting a blog		Blogging about social difficulties in open response blog; blogging about social difficulties (closed responses; n=27); blogging about social difficulties (open responses; n=26); blogging about general subjects (open responses; n=28); blogging about general subjects (closed responses; n=27); writing a private diary about social difficulties (n=26); MC (n=27)			Web; pre-post					Blogging about social-emotional difficulties improved writer’s social-emotional condition (ratings by independent judges on the Judgment of social-emotional condition) Improvements in the Rosenberg Self-Esteem Scale [69] (Cohen d=0.211; *P*=.001), Interpersonal Activities Checklist, and Index of Peer Relationship [70] compared with other blogging groups Social-emotional difficulty blogs open to public responses had improved judge-rated outcomes Findings remained stable at the 2-month follow-up
	Sportel et al [60]; CBMT^o^	N=240 (66); 13-15 (14.1)		>10 RCADS social phobia; (girls)>9 (boys)		Attentional bias modification tasks, strengthening the association between social-evaluative situations and positive outcomes, enhancing implicit self-esteem (n=86); AC (n=84); MC (n=70)			Web; RCT					CBMT showed greatest improvements (Single Target Implicit Association Test, Adolescent Interpretation and Belief Questionnaire) CBT and CBMT showed significant improvements after the test for social RCADS (Cohen d=0.051; *P*=.001) and test anxiety (Spielberger Test Anxiety Inventory [71]), significantly stronger for the CBT group Follow-up scores suggest effects remained at 12 months, with Cognitive Bias Modification showing lower negative associations than other groups

^a^SPARX: Smart, Positive, Active, Realistic, X-factor thoughts [50].

^b^CDRS-R: The Children’s Depression Rating Scale-revised [72].

^c^CBT: cognitive behavioral therapy.

^d^RCT: randomized controlled trial.

^e^MFQ: Moods and Feelings Questionnaire [61].

^f^SCAS: Spence Children’s Anxiety Scale [73].

^g^WL: wait-list.

^h^RADS: Reynolds Adolescent Depression Scale [74].

^i^RCADS: Revised Child Anxiety and Depression Scale [75].

^j^MC: monitoring control.

^k^AC: attentional control.

^l^Subgroup analysis: numbers are presented so that one can see how many of the same were included in the subgroup analysis.

^m^Missing data.

^n^CES-D: Centre for Epidemiological Studies Depression Scale [76].

^o^CBMT: cognitive bias modification training [60].

### Descriptions of Interventions

In total, this review found papers on 8 different interventions (*SPARX*, *The Journey*, *MoodGYM*, *Mobiletype, Shamiri-Digital*, *Stressbusters,* blogging, and CBMT) delivered by mobile phones, the internet, and computers; see Table 1 for an overview of the interventions and their components. Of the 3 interventions that were freely available, 2 (67%) could be used worldwide (blogging and *Shamiri-Digital*), and 1 (33%) was restricted to those living in the country where it was developed (*SPARX*). A more detailed description of the content of the interventions is provided in Multimedia Appendix 4 [49,50,52-60]. One of the interventions required an annual fee (not required by participants in this study; *MoodGYM*), and 4 of the interventions were available only through research institutes (*Mobiletype*, *Stressbusters,* CBMT, and *The Journey*). Professional contact during these self-help interventions was mostly classroom supervision, whereas young people completed the task and site visits to check safety concerns. The length of interventions varied from 1 to 28 sessions, with the duration of sessions ranging between 3 and 60 minutes. All studies had a follow-up, with the shortest being 4 weeks and the longest at 12 months. All the studies had a control intervention, as shown in Table 1.

### Outcome Measures

Outcome measures, both primary and associated, across interventions were highly heterogenous, with a variety of scales used to assess clinical symptoms or psychological and social well-being. The most commonly used outcome measures across the included studies reflected 2 mental health conditions: anxiety and depression. The most commonly used scale was the Revised Child Anxiety and Depression Scale, which was included across 4 of the 6 studies aimed at improving depression [49-52]. A total of 3 studies used the Mood and Feelings Questionnaire [50,57,58], the Children’s Depression Rating Scale-revised [49,50,54], and Spence Children’s Anxiety Scale [49,50,57], and 2 studies used the Center for Epidemiological Studies Depression Scale [53,54] across interventions aimed at depression and anxiety and depression. Furthermore, outside of measures of anxiety and depression, the Pediatric Quality of Life Enjoyment and Satisfaction Questionnaire, a measure of quality of life, was used by 3 interventions for improving depression [49,50,52]. The Rosenberg Self-Esteem Scale, a measure of self-esteem, was used in 3 separate studies for interventions aimed at improving anxiety and depression and social functioning [53,54,59], and the Adolescent Coping Scale-Short to rate coping was used in interventions for both depression and anxiety interventions [52,56]. In total, there were 36 different types of outcome measures, 28 of which were used only by individual studies.

Owing to a few studies using the same or similar outcome measures (ie, across all interventions, 3 studies had no depression outcomes and those that did measure depression used a variety of scales), 2 studies reporting on the same data set, skewed data, and moderate to high levels of potential bias across the studies, it was decided that conducting a meta-analysis would not reflect the data appropriately, and thus, a meta-analysis was not undertaken.

### Characteristics of Participants

In total there were 1460 participants, ranging from 11 to 24 years, with a mean average of 15.4 years (for a given average age, weighted by sample size). On starting the intervention, a clinical threshold on a questionnaire for anxiety or mood disorder was used by nearly all the studies other than the study by Boniel-Nissim and Barak [59], who used an index of peer relationships, that is, a well-being measure rather than a clinical measure. Subgroup analyses from 3 studies with school-based populations showed elevated scores for depression symptoms [53,54] and one with elevated anxiety and depression [55].

### Risk of Bias Within and Between Studies

Of the 7 standard quality criteria, 7 studies met 4 or more of the 7 criteria, deeming them to have moderate to low risk of bias [50,52,53,55,56,59,60]. No studies met 6 or more standard quality criteria. Of the remaining studies meeting less than half of the criteria, 4 studies met 3 of the criteria [49,51,57,58], and the study by O’Kearney et al [54] only met 1 of the criteria. Most studies (10/12, 83%) applied adequate random sequence allocation, which is expected to create comparable groups through randomization that could be replicated. Fewer studies (6/12, 50%) applied group allocation without any prior knowledge of the participants, which increased the risk of groups being selected for or against using an intervention. There was a similarly high risk of performance bias, where participants were not blind to the intervention (7/12, 58%). Less than half of the studies used blinded outcome assessors (7/12, 58%), leading to potential detection bias. A total of 3 studies published a protocol that was available on the web before publishing their papers; therefore, there was insufficient information to detect reporting bias. Attrition bias was of higher quality across studies, with most studies reporting the flow of participants throughout the process (10/12, 83%). In *other biases* the highest risk was found, as almost all the studies displayed experimenter bias, whereby the intervention developer was the author of the study (11/12, 92%).

Trial retention varied from a very high score of 100% to a very low score of 10.4% (Table 2). Similar variations were seen in intervention retention scores, from 100% of the sample reported to have completed the full intervention to 0.04% completing all modules of the intervention. Outlier analysis indicated that the very low scores obtained in the study by Lillevoll et al [53] were outliers in an outlier analysis based on postintervention trial retention (95% CI 53-115). Not including the study by Lillevoll et al [53], across studies of a total of 1256 participants, 1071 were retained giving an average trial retention of 85.2%. At follow-up, for the studies who provided the information, the range of retention varied from 57.9% to 100%. Of the 3 school-based populations using a universal intervention, with pooled subgroup analysis, 2 (67%) showed trial and intervention retention rates at the lower end of the scale [53,54], whereas 1 (33%) showed the highest trial retention, retaining all young people who had elevated depression throughout the follow-up [55]. The CBMT also showed low levels of intervention retention [60]. One study did not provide sufficient details to calculate intervention retention [59]. Looking at trial retention compared with intervention retention within trials, the rates had an average difference of 12.4 %.

**Table 2:** A table of the numbers and percentages of participants enrolled in the studies at baseline, poststudy, and follow-up to display rates of retention, in descending order, with an amount of professional contact. Percentages for participants retained were calculated by taking the total number of participants at baseline and dividing it by the participants at poststudy or follow-up. These scores were used to calculate the overall mean and median percentages.

Author	Professional contact	Participants at baseline, n	Trial retention, n (%)			Intervention retention (%)
			After the intervention	Follow-up		
Osborn et al [55]^a^	Shamiri-digital was completed in classrooms over 1 session, where teachers supervised.	56	56 (100)	56 (100)	100	
Fleming et al [49]	During class time under minimal supervision from school staff. Weekly check-ins from researcher for safety checks and practical support.	32	31 (96.8)	25 (78.1)	69	
Poppelaars et al [51]	Completed at home.	208	201 (96.6)	159 (76.5)	93 (self-report)	
Merry et al [50]	Safety checks at all time points plus an additional check at 1 month.	187	170 (90.9)	168 (89.8)	60	
Smith et al [58]	Study information given by a clinical psychologist before enrollment. Stressbusters completed at school in an assigned room.	112	100 (89.2)	99 (88.4)	86	
Stasiak et al [52]	School counselors instructed to give practical support. Therapeutic support provided only if requested from young people. Completed in the counselor’s office.	34	30 (88.2)	25 (73.5)	82	
Sportel et al [60]	Weekly emails with links to complete cognitive bias modification training and a reminder if not completed.	240	200 (83)	139 (57.9)	42	
Boniel-Nissim and Barak [59]	Email checks conducted for diary entries.	161	124 (78)	—^b^	—	
Reid et al [56]	High-risk alert activated by psychologist if a young person indicated suicide or self-harm; young person’s community team informed.	112	87 (77.6)	86 (76.7)	52.9	
O’Kearney et al [54]^a^	Completed at school during tutor period under teacher supervision. Researchers attended the first session to check successful log-in.	23	17 (73.9)	18 (72)	40 (half or more modules)	
Wright et al [57]	Practical support to accessing Stressbusters from researchers.	91	55 (60.4)	No follow-up data	70	
Lillevoll et al [53]^a^	Automated email reminders of level of MoodGYM use (tailored email group not included).	198	19 (10.4)	No follow-up data	0.04	

^a^Studies using subgroup analysis data only and not the original sample number.

^b^Data not available.

### Self-help Interventions for Depression or Depressive Symptoms

Of the 3 interventions for depression, *SPARX* and *The Journey* were delivered by an offline computer program, and *MoodGYM* was accessed through the internet. All the interventions consisted of key components of CBT, which included cognitive restructuring, problem-solving, relaxation training, social and communication training, and practice. Other components involved in the interventions included psychoeducation (*SPARX* and *MoodGYM*) or explicit homework tasks to reflect on learned skills and apply them to real life (*SPARX*).

Self-help interventions aimed at depressive symptoms for young people showing that symptoms of depression were effective when using fantasy-world games: *The Journey* and *SPARX* [49-52]. These programs were found to reduce depression after the intervention, and improvements were maintained at the 3-month [50] and 1-year follow-up [51]. Furthermore, of these 4 studies, at least 88% of their participants completed postintervention outcome measures and 73% completed follow-up.

Other self-help intervention studies aimed at reducing depressive symptoms have used *MoodGYM*, a traditional linear-style approach of computerized CBT [53,54]. O’Kearney et al [54] found risk and vulnerability reduction for depression after the intervention and at follow-up. These findings were not supported by the study by Lillevoll et al [53], whose sample had high attrition rates of nearly 70% and reported no significant changes, likely because of loss of power. Although the study by O’Kearney et al [54] did show trends toward improvement in depressive symptoms, both studies had small final sample sizes and showed a high risk of bias.

### Self-help Interventions for Depression and Anxiety

Three interventions were found for anxiety and depression: *Mobiletype* [56], *Shamiri-Digital* [55], and *Stressbusters* [57,58]. *Mobiletype* is delivered through a mobile phone using an app that self-monitors to indicate to general practitioners and patients whether there is a need for greater intervention. The results suggest that the use of self-monitoring for young people with depression and anxiety symptoms improves emotional self-awareness. These results are promising given that the control intervention was an attentional control similar to the intervention, and the study scored highly for a low risk of bias. Furthermore, retention rates in the trial were good considering that this intervention was relatively demanding, requiring participants to make entries 5 times a day over 2 to 4 weeks, with results being maintained at the 6-week follow-up.

*Shamiri-Digital* is delivered through a web browser via a PC and includes a single component which aims to be brief and requires minimal training to deliver. The intervention involves components related to well-being rather than clinical components, such as growth mindset, gratitude, and value affirmations. The results are promising as the attentional control mirrored the structure and was similar to the Shamiri intervention but focused on note-taking and effective study. In addition, retention in the trial was very successful, as all participants completed the trial and a 2-week follow-up. This study showed a fairly low risk of bias, with a high risk only seen in selective reporting and the use of intervention developers evaluating the program.

*Stressbusters* is a CBT-based offline computer intervention that uses a traditional linear-style approach to teach how to recognize and challenge emotions and thoughts. A total of 2 studies showed a reduction in scores for anxiety and depression after the intervention and at the 4- and 6-month follow-up; however, in the study by Wright et al [57], this was not significant compared with the control group, who also showed improvement. Furthermore, there was nearly a 30% difference between the rates of attrition for both studies with the study by Wright et al [57] having 60.4% remaining in their sample at follow-up, compared with 89.2% for the study by Smith et al [58]. Finally, both studies scored poorly with a high or unknown risk of bias across the 4 criteria for each study, in particular, relating to allocation concealment, blinding of participants and researchers, and a lack of independent researchers conducting the research.

### Social Functioning

Boniel-Nissim and Barak [59] found improved outcomes when blogging about social difficulties and greater improvements when the blog was open to peers to respond to measures of social-emotional distress and social behavior. These findings were retained at the follow-up and showed a low risk of bias. The study by Sportel et al [60] used CBMT to modify the negative interpretations of people with social anxiety. This intervention reduced the negative associations for up to 12 months after the intervention. Although the CBT control group showed greater improvements than the CBMT group, the CBMT group showed greater improvements compared with the no intervention control group. A low risk of bias was also associated with this intervention.

### Well-being Outcomes Not Related to the Initial Aim of the Intervention

Across studies, the reported effects were not always limited to those associated with the main aim of the intervention. Of the 2 studies exploring *SPARX* for depression, one study found improvements in scales for hopelessness and anxiety, alongside improvements in depression scales [50]. *The Journey* succeeded in improving depression scores while also showing improvements in problem-solving skills for coping [52]. As anticipated, one of the *MoodGYM* studies showed patterns toward improvements in depression and vulnerability to depression but also found improved self-esteem and attitudes toward depression, although none of these reached significance [54], and this was not matched by the other *MoodGYM* study [53]. In *Mobiletype* users, although a significant increase in depression and anxiety was not found, a general trend toward improvement was noted, and a significant increase in emotional self-awareness compared with control groups was shown [53]. In the study by Smith et al [58] on *Stressbusters*, a significant increase in school attendance was also seen alongside improvements in anxiety and depression scores. Blogging about social difficulties improved outcomes in social dimensions such as social-emotional distress and engagement in social behaviors and self-esteem [59]. The CBMT group showed improvements in implicit associations and social interpretations over the other groups. Both the CBMT and CBT groups showed reductions in social phobia and test anxiety [60].

## Discussion

### Summary of Evidence

This review examined the effectiveness of self-help interventions aimed at improving psychological well-being in young people with low psychological well-being, identified through self-selection of perceived poor well-being, clinical diagnosis of anxiety or mood disorders, or meeting a threshold suggestive of mental health symptoms. A total of 12 studies met the search criteria, 10 (83%) of which were RCTs and 2 (17%) were controlled pre-post studies. Most interventions [49-52,56-58] were aimed at young people who showed elevated symptoms indicative of depressive or mood disorders on a symptom rating scale, including interventions aimed at both anxiety and depression. One study focused on those displaying elevated symptoms of anxiety using anxiety symptom rating scales [60], and the other included young people who scored below average within the sample on a social functioning scale [59]. This was the only study to use a measure of well-being which was not also a clinical measure, that is, depression or anxiety. A total of 3 studies were universal interventions for school-based populations, with subgroup analysis of young people showing elevated depression [53,54] and anxiety and depression [55]. No studies included young people who had self-perceived reduced well-being or a diagnosis of a mental health condition as inclusion criteria. Overall, the narrative evaluation indicated that predominately or entirely self-administered self-help interventions improved psychological well-being in the areas intended for intervention.

The results also highlighted that such interventions can lead to improvements in other areas associated with psychological well-being, such as self-esteem, emotional self-awareness, and problem-solving skills, which had not been the primary outcome of the intervention. The studies indicated reasonable levels of trial retention, with over three-fourths of participants being retained after the intervention for 9 of the 12 studies and at follow-up for half of the studies. Furthermore, the intervention retention levels were promising, as they suggested that young people completed over half of the intervention. Although the relationship between trial retention (ie, completing all outcome measures of a trial) and intervention retention (ie, completing all intervention modules) is not the same, the results seem to support the notion that intervention and trial retention follow a similar relationship in terms of indicating the level of young people’s engagement [77].

Most of the included studies provided sufficient details to calculate trial (12/12, 100%) and intervention retention rates (11/12, 92%). Often, these data were not readily available, and sometimes this was not available at all for follow-up data (11/12, 92%), even though participant flowcharts are recommended for transparent reporting of RCTs [78]. For example, a participant may be regarded as a “completer,” because they completed all the questionnaires in the study, but this does not ascertain whether they completed all the modules or active time spent in the intervention. Trial settings are believed to consist of *push factors* that influence attrition rates, with more *open* or pragmatic trials tending to have greater dropout [77]. A sample in the study by Lillevoll et al [53] showed notably reduced adherence compared with other studies, possibly as a result of running a trial in a more naturalistic setting. Therefore, it is highly recommended that future interventions, studies, and systematic reviews provide details on both intervention and trial retention. Nonetheless, the findings presented here suggest that young people engage well in self-help intervention trials.

The greatest promise for self-help digital interventions seemed to be CBT-based fantasy-style games (eg, *SPARX* and *The Journey)* for reducing symptoms of depression, which demonstrated good trial retention and follow-up rates, whereas traditional linear-style interventions showed greater variation in improvement (eg, *MoodGYM)*. Interventions used by young people with anxiety and depressive symptomatology showed general improvements in well-being, with mood monitoring (*Mobiletype*) leading to the maintenance of increased emotional self-awareness at follow-up. This was similar for linear-style interventions with one of the *Stressbusters* studies and the *Shamiri-Digital* trial, reaching significance for reductions in depressive symptoms. Finally, interventions aimed at improving social functioning were effective after the intervention and remained significantly so at follow-up for the blogging group. Some of the seemingly less complex interventions (*Shamiri-Digital* and blogging) are freely available and open source, which could present interventions that are truly accessible to young people, raising an important point about the need for available and effective interventions. Previous reviews have suggested that the use of guidance within self-help interventions improves the magnitude of the effect [16,79], but these findings suggest that stand-alone interventions can support young people with elevated symptoms or social functioning difficulties without the need for therapist assistance. Self-administered therapies have major advantages to guided self-help because of their scalability, although not necessitating further demands from already overburdened services, including cost, location, and trained professionals [80,81].

A meta-analysis of the studies included in this report was not deemed appropriate because of a lack of high-quality studies, as nearly half of the papers met less than half of the quality criteria required to protect against biases. Notably, 92% (11/12) of studies demonstrated experimenter bias, and only 8% (1/12) of studies were identified as having a low risk of bias for selective reporting. Conducting a meta-analysis with biased data can lead to misleading conclusions [82]. For example, inappropriate concealment of participant allocation or selection bias, as found in more than half of the studies included in this review, can lead to 30% overestimation of the treatment effect [83]. Although there is a need for more high-quality assessments of self-help interventions, the time frame around running an empirical study against the delivery time of commercial interventions makes this difficult. As of March 2021, there are 53,979 and 53,054 medical health apps available in the Apple App Store and Google Play, respectively, which have shown increases of 4.86% and 6.51%, respectively, since the last quarter of 2020 [84,85]. In light of the long time it takes to complete and publish RCTs, it is unrealistic for researchers to complete controlled assessments of interventions with the unprecedented rate of growth of apps and digital technology development [34,86]. Consequently, the use of RCTs to assess research has been questioned, with iterative approaches to development proposing that RCTs should be used only to assess the overall functionality of an intervention or its theoretical basis rather than for minor intervention modifications [86,87]. For these reasons, this review recommends conducting higher quality controlled before and after studies that do not overlook the bias criteria. This suggestion also meets the National Institute for Health and Care Excellence best practice standards for self-management of DHIs, requiring high-quality studies with a comparison group [88]. One crucial method to improve quality calls for researchers outside the developing group of interventions to carry out controlled trials to minimize experimenter bias. Furthermore, the inclusion of non-RCT studies in future systematic reviews will enable the evaluation of the most recent interventions to provide a comprehensive overview of whether self-help interventions can effectively support young people with mental disorders.

Despite a broad literature search, most trials were interested in young people with clinical symptoms rather than reduced psychological well-being, which was also mirrored in the outcomes of the trials. This highlights the lack of recognition of the importance of well-being in achieving positive mental health outcomes. In contrast, self-help interventions were fairly narrowly aimed at young people with mood, anxiety, or social difficulties, and none were found for other underexplored disorders such as neurodevelopmental, obsessive-compulsive, sleep, eating, and anger conditions. Those living with such conditions would likely benefit from self-help interventions to support well-being difficulties related to the clinical symptomology of their diagnosis or because of living with their condition [4]. As mentioned earlier, this review calls for higher quality-controlled studies on self-help interventions with populations of young people with such disorders and includes measures of psychological well-being in the sample criteria and outcome measures. Nevertheless, considering the findings of Bennett et al [16], parent-involved self-help interventions may be more useful than predominantly self-administered interventions; the inclusion of parental involvement alongside self-help interventions may overcome some of the aforementioned barriers to interventions while retaining young person engagement, compliance, and increasing the effectiveness of the intervention. This is in line with evidence that parent-involved multimedia and bibliotherapy interventions are as beneficial for behavioral disorders as therapist-led interventions in the long term [89] and that parent involvement within CBT interventions is effective for adolescent anxiety disorders [90]. This area of research warrants further investigation as the digital delivery of parent training could facilitate access to these interventions.

### Limitations

This review had several limitations. A small number of studies matched the inclusion criteria, and only 1 intervention was tested with those experiencing reduced psychological well-being outside of clinically diagnosed illnesses (ie, anxiety and depression). This could be because of the specific focus on psychological and social well-being, not including outcome measures related to the cognitive, economic, and physical domains of well-being [42]. Although the authors recognize that these domains relate to and are important aspects of a young person’s well-being, psychological and social functioning were felt to be the most impaired when living with perceived reduced well-being or a diagnosed mental health condition, and identifying interventions aimed at improving these aspects was a priority. By including other aspects of well-being, it is possible that more interventions would have been found. As this review only considered common childhood disorders, conditions such as psychosis were not included, although it is recognized that children with uncommon conditions would also experience reduced well-being. Therefore, these results cannot be generalized to less common disorders that were not included in this review. The included studies were mainly developed by and run in societies with a Western cultural influence, meaning that few of the findings can be generalized to other cultures. Finally, data were missing from certain studies which affected study inclusion, conclusions that could be drawn for retention, and the ability to conduct a meta-analysis. Although the authors were contacted to request information related to the eligibility criteria, a lack of response meant that they could not be included. Alongside narrowing the search to English studies on the web and including quantitative studies only, this may have reduced the pool of the data set.

### Conclusions

The findings of this review support the utility of digital self-help interventions for young people with elevated symptoms of depression and anxiety. Only 2 interventions were identified for young people experiencing social functioning difficulties; therefore, it is not possible to generalize the findings to understand whether self-help interventions improve well-being in those experiencing reduced well-being. Interventions were found to be effective and had reasonable levels of retention, suggesting that they were acceptable for youth populations. The greatest promise seems to lie in fantasy-style interventions for young people who experience symptoms of depression. Nonetheless, traditional linear-style interventions were still beneficial to users with depression and anxiety symptoms and social dysfunction. The collective advantages of self-administered self-help interventions included low costs, ease of access, and reduced need for trained professionals, but simple interventions were deemed to be an especially feasible option in helping overcome barriers to accessing mental health care for youth populations, as some of these were also readily available to access. Greater efforts are warranted to improve the quality of studies, and greater consensus is required on the use of outcome measures in relation to retention and adherence, as well as different aspects of well-being, to help determine the impact of these interventions on broader well-being and in *real-world* settings. Specifically, further research in this area should focus on improving the quality of studies within the research of predominantly or entirely self-administered digital interventions for young people who show elevated symptomatology of mental health problems and reduced psychological well-being, and have mental, behavioral, and neurodevelopmental disorders. With the improved quality of studies, a meta-analysis could be performed which would provide more precise indicators of the effectiveness of self-help interventions. Research should also explore the use of alternatives to RCTs in the assessment of digital interventions to help close the gap between the progression of technology and the dissemination of empirical studies.

## Appendix

Multimedia Appendix 1 Population, intervention, comparators, and outcomes table.

Multimedia Appendix 2 Example search strategy for MEDLINE database.

Multimedia Appendix 3 Reference list of hand searched systematic reviews.

Multimedia Appendix 4 Detailed description of intervention content.

## Abbreviations

CBMT: cognitive bias modification training
CBT: cognitive behavioral therapy
DHI: digital health intervention
PRISMA: Preferred Reporting Items for Systematic Reviews and Meta-Analyses
RCT: randomized controlled trial
