# Supplementary material for: Self-help Digital Interventions Targeted at Improving Psychological Well-being in Young People With Perceived or Clinically Diagnosed Reduced Well-being: Systematic Review
Source: JMIR Ment Health. 2022 Aug 26;9(8):e25716. doi: 10.2196/25716 (PMC9463613; doi:10.2196/25716)
Supplement: Multimedia Appendix 1 [file mental_v9i8e25716_app1.docx]

**Appendix 1: PICO table adapted for different databases**

| **Population** | **Condition** | **Intervention Mode** | **Intervention Type** |
| --- | --- | --- | --- |
| Adolescen* | Neurodevelopmental Disorders (MeSH) | E?health | Self?help |
| Teen* | Neurocognitive Disorders (MeSH) | Internet intervention* | Self?management |
| Youth* | Tic Disorders (MeSH) | Internet | Self?care |
| Minor* | Tics (MeSH) | Online intervention* | Self-help groups (MeSH) |
| Child* | Stereotyped behaviour (MeSH) | Mobile application* | Self-management (MESH) |
| Young pe* | Tourette Syndrome (MeSH) | Computer Intervention* | Self-care (MeSH) |
| Adolescent (MeSH) | Obsessive-Compulsive Disorder (MeSH) | Tablet intervention* |  |
| Child (MeSH) | Obsessive Behavior [mh] | Electronic?Health |  |
|  | Compulsive Personality Disorder (MeSH) | Digital?health intervention* |  |
|  | Neurotic disorders (MeSH) | Digital Health |  |
|  | Autistic Disorder (MESH) | Digital intervention* |  |
|  | Autism Spectrum Disorder (MESH) | Electronic intervention* |  |
|  | Asperger Syndrome (MESH) | Telemedicine (MeSH) |  |
|  | Attention deficit disorder with hyperactivity (MESH) | Computer assisted instruction (MESH) |  |
|  | Impulsive behaviour (MESH) | Therapy, Computer-Assisted (MESH) |  |
|  | Attention deficit and disruptive behaviour disorders (MESH) | Medical Informatics Applications (MeSH) |  |
|  | Substance-related Disorders (MeSH) | Computer Systems (MeSH) |  |
|  | Anxiety (MeSH) | Telephone (MeSH) |  |
|  | Anxiety Disorders (MeSH) | Wireless Technology (MeSH) |  |
|  | Stress, psychological (MeSH) |  |  |
|  | Stress, Physiological (MESH) |  |  |
|  | Mood Disorders (MeSH) |  |  |
|  | Mental Disorders (MESH) |  |  |
|  | Affective symptoms (MESH) |  |  |
|  | Depression (MESH) |  |  |
|  | Depressive Disorder (MeSH) |  |  |
|  | Affect (MESH) |  |  |
|  | Child Behavior Disorders [mh] |  |  |
|  | Feeding and Eating Disorders [mh] |  |  |
|  | Attitude to health (MESH) |  |  |
|  | “Quality of Life” (MESH) |  |  |
|  | Sleep Wake Disorders (MeSH) |  |  |
|  | Self-Injurious Behavior (MeSH) |  |  |
|  | Fatigue (MeSH) |  |  |
|  | Rage (MeSH) |  |  |
|  | Involuntary Movement* |  |  |
|  | Coprolalia |  |  |
|  | Copropraxia |  |  |
|  | Echolalia |  |  |
|  | Echopraxia |  |  |
|  | Palilalia |  |  |
|  | Palipraxia |  |  |
|  | Premonitory Urge |  |  |
|  | Premonitory Urges |  |  |
|  | Coprophenomena |  |  |
|  | Echophenomena |  |  |
|  | Paliphenomena |  |  |
|  | “Chronic motor or vocal tic disorder*” |  |  |
|  | Impuls* |  |  |
|  | Compulsi* |  |  |
|  | Obsessive Behaviour |  |  |
|  | Obsessive Compulsive Symptom* |  |  |
|  | Hyperactiv* |  |  |
|  | Hyperkine* |  |  |
|  | Hyper?activ* |  |  |
|  | Conduct Disorder* |  |  |
|  | Anti?social behaviour* |  |  |
|  | Depression |  |  |
|  | Anxiety |  |  |
|  | Anxiety Disorder* |  |  |
|  | Stress |  |  |
|  | Affective Disorder* |  |  |
|  | Well-being |  |  |
|  | Mood |  |  |
|  | Coping |  |  |
|  | Distress |  |  |
|  | Sleep Disturbance* |  |  |
| **AND** | **AND** | **AND** | **AND** |
|  |  |  |  |
