# Supplementary material for: Self-help Digital Interventions Targeted at Improving Psychological Well-being in Young People With Perceived or Clinically Diagnosed Reduced Well-being: Systematic Review
Source: JMIR Ment Health. 2022 Aug 26;9(8):e25716. doi: 10.2196/25716 (PMC9463613; doi:10.2196/25716)
Supplement: Multimedia Appendix 2 [file mental_v9i8e25716_app2.docx]

**Appendix 2: Example search strategy for Medline**

| **Name of the database** | **Medline** |  |  |  |  |
| --- | --- | --- | --- | --- | --- |
| **Platform** | **OvidSP** |  |  |  |  |
| **Date of Search** | **24.09.21** |  |  |  |  |
| **Database Coverage** | **1946 present** |  |  |  |  |
| **Search Strategy** |  | **Population** | **Condition** | **Intervention Mode** | **Intervention Type** |
|  | **OR** | ((Adolescen* or Teen* or Youth* or Minor* or Child* or Young pe*).ti. or (Adolescen* or Teen* or Youth* or Minor* or Child* or Young pe*).ab. or Adolescent.sh. or Child.sh.) | (Coprolalia or Copropraxia or Echolalia or Echopraxia or Palilalia or Palipraxia or Premonitory Urge* or Coprophenomena or Echophenomena or Paliphenomena or "Chronic motor or vocal tic Disorder*" or Impuls* or Compulsi* or Obsessive Compulsive Symptom* or Obsessive Behavio?r* or Hyperactiv* or Hyperkine* or Hyper?activ* or Conduct Disorder* or Anti?social behaviour* or Depression or Anxiety or Anxiety Disorder* or Stress or Affective Disorder* or Mood or Well?being or Coping or Distress or Sleep Disturbance* or Involuntary movement*).ti. | (E?health or Internet intervention* or Internet or Online intervention* or Mobile application* or Computer Intervention* or Tablet intervention* or Electronic?Health or Digital?intervention or Digital?health intervention* or Digital?Health or Electronic intervention*).ti or (E?health or Internet intervention* or Internet or Online intervention* or Mobile application* or mobile intervention* or Computer Intervention* or Tablet intervention* or Electronic?Health or Digital?intervention or Digital?health intervention* or Digital?Health or Electronic intervention*).ab or Telemedicine.sh. or Computer assisted instruction.sh. or Therapy, Computer-Assisted.sh. or Medical Informatics Applications.sh. or Computer Systems.sh. or Telephone.sh. or Wireless Technology.sh. | (Self?help or Self?management or Self?care).ti. or (Self?help or Self?management or Self?care).ab. or Self-help groups.sh. or Self-management.sh. or Self-care.sh. |
|  | **OR** |  | (Coprolalia or Copropraxia or Echolalia or Echopraxia or Palilalia or Palipraxia or Premonitory Urge* or Coprophenomena or Echophenomena or Paliphenomena or "Chronic motor or vocal tic Disorder*" or Impuls* or Compulsi* or Obsessive Compulsive Symptom* or Obsessive Behavio?r* or Hyperactiv* or Hyperkine* or Hyper?activ* or Conduct Disorder* or Anti?social behaviour* or Depression or Anxiety or Anxiety Disorder* or Stress or Affective Disorder* or Mood or Well?being or Coping or Distress or Sleep Disturbance* or Involuntary movement*).ab. |  |  |
|  | **OR** |  | Neurodevelopmental Disorders.sh. or Neurocognitive Disorders.sh. or Tic Disorders.sh. or Tics.sh. or Stereotyped behaviour.sh. or Tourette Syndrome.sh. or Obsessive-Compulsive Disorder.sh. or Obsessive Behavior.sh. or Compulsive Personality Disorder.sh. or Neurotic Disorders.sh. or Autistic Disorder.sh. or Autism Spectrum Disorder.sh. or Asperger Syndrome.sh. or Attention deficit disorder with hyperactivity.sh. or Impulsive behaviour.sh. or Substance-related Disorders.sh. or Anxiety.sh. or Anxiety Disorders.sh. or Mood Disorders.sh. or Stress, psychological.sh. or Stress, Physiological.sh. or Mental Disorders.sh. or Affective symptoms.sh. or Depression.sh. or Depressive Disorder.sh. or Affect.sh. or Child Behavior Disorders.sh. or Attitude to health.sh. or Sleep Wake Disorders.sh. or Self-Injurious Behavior.sh. or Fatigue.sh. or Rage.sh. |  |  |
|  | **OR** |  | “Attention deficit and disruptive behavior disorders”.sh. | *NOTE must be added seperately into the search as MESH headings |  |
|  | **OR** |  | “Feeding and Eating Disorders”.sh | *NOTE must be added seperately into the search as MESH headings |  |
|  | **OR** |  | “Quality of life”.sh | *NOTE must be added seperately into the search as MESH headings |  |
|  | **OR** |  |  | *NOTE must be added seperately into the search as MESH headings |  |
|  |  | **AND** | **AND** | **AND** | **AND** |
