# Supplementary material for: Self-help Digital Interventions Targeted at Improving Psychological Well-being in Young People With Perceived or Clinically Diagnosed Reduced Well-being: Systematic Review
Source: JMIR Ment Health. 2022 Aug 26;9(8):e25716. doi: 10.2196/25716 (PMC9463613; doi:10.2196/25716)
Supplement: Multimedia Appendix 3 [file mental_v9i8e25716_app3.docx]

**Appendix Three: References searched and added to the review**

| Ahmead, M., & Bower, P. (2008). The effectiveness of self help technologies for emotional problems in adolescents: A systematic review. *Child and Adolescent Psychiatry and Mental Health*, *2*. https://doi.org/10.1186/1753-2000-2-20 |
| --- |
| Bennett, S. D., Cuijpers, P., Ebert, D. D., McKenzie Smith, M., Coughtrey, A. E., Heyman, I., Manzotti, G., & Shafran, R. (2019). Practitioner Review: Unguided and guided self-help interventions for common mental health disorders in children and adolescents: a systematic review and meta-analysis. *Journal of Child Psychology and Psychiatry and Allied Disciplines*, *8*, 828–847. https://doi.org/10.1111/jcpp.13010 |
| Donker, T., Petrie, K., Proudfoot, J., Clarke, J., Birch, M. R., & Christensen, H. (2013). Smartphones for smarter delivery of mental health programs: A systematic review. *Journal of Medical Internet Research*, *15*(11), 1–13. https://doi.org/10.2196/jmir.2791 |
| Ebert, D., Zarski, A. C., Christensen, H., Stikkelbroek, Y., Cuijpers, P., Berking, M., & Riper, H. (2015). Internet and computer-based cognitive behavioral therapy for anxiety and depression in youth: A meta-analysis of randomized controlled outcome trials. *PLoS ONE*, *10*(3), 1–15. https://doi.org/10.1371/journal.pone.0119895 |
| Fleming, T., Cheek, C., Merry, S. N., Thabrew, H., Bridgman, H., Stasiak, K., Shepherd, M., Perry, Y., Hetrick, S., & Fleming, T. (2014). Serious games for the treatment or prevention of depression: a systematic review. *Revista de Psicopatología y Psicología Clínica*, *19*(193), 227–242. https://doi.org/10.5944/rppc.vol.19.num.3.2014.13904 |
| Grist, R., Porter, J., & Stallard, P. (2017). Mental Health Mobile Apps for Preadolescents and Adolescents: A Systematic Review. *Journal of Medical Internet Research*, *19*(5), e176. https://doi.org/10.2196/jmir.7332 |
| Hollis, C., Falconer, C. J. C., Martin, J., Whittington, C., Stockton, S., Glazebrook, C., & Davies, E. B. E. (2016). Annual Research Review: Digital health interventions for children and young people with mental health problems: a systematic and meta-review. *Journal of Child Psychology and Psychiatry*, *58*(4), 474–503. https://doi.org/10.1111/jcpp.12663 |
| Pennant, M. E., Loucas, C. E., Whittington, C., Creswell, C., Fonagy, P., Fuggle, P., Kelvin, R., Naqvi, S., Stockton, S., & Kendall, T. (2015). Computerised therapies for anxiety and depression in children and young people: A systematic review and meta-analysis. *Behaviour Research and Therapy*, *67*, 1–18. https://doi.org/10.1016/j.brat.2015.01.009 |
| Podina, I. R., Mogoase, C., David, D., Szentagotai, A., & Dobrean, A. (2016). A Meta-Analysis on the Efficacy of Technology Mediated CBT for Anxious Children and Adolescents. *Journal of Rational - Emotive and Cognitive - Behavior Therapy*, *34*(1), 31–50. https://doi.org/10.1007/s10942-015-0228-5 |
| Rooksby, M., Elouafkaoui, P., Humphris, G., Clarkson, J., & Freeman, R. (2015). Internet-assisted delivery of cognitive behavioural therapy (CBT) for childhood anxiety: Systematic review and meta-analysis. *Journal of Anxiety Disorders*, *29*(1), 83–92. https://doi.org/10.1016/j.janxdis.2014.11.006 |
| Schleg, S., Bürger, C., Schmidt, L., Herbst, N., Voderholzer, U., Schlegl, S., Bürger, C., Schmidt, L., Herbst, N., & Voderholzer, U., Schlegl, S., Bürger, C., Schmidt, L., Herbst, N., & Voderholzer, U. (2015). The Potential of Technology-Based Psychological Interventions for Anorexia and Bulimia Nervosa: A Systematic Review and Recommendations for Future Research. *Journal of Medical Internet Research*, *17*(3), e85. https://doi.org/10.2196/jmir.3554 |
