# Supplementary material for: Self-help Digital Interventions Targeted at Improving Psychological Well-being in Young People With Perceived or Clinically Diagnosed Reduced Well-being: Systematic Review
Source: JMIR Ment Health. 2022 Aug 26;9(8):e25716. doi: 10.2196/25716 (PMC9463613; doi:10.2196/25716)
Supplement: Multimedia Appendix 4 [file mental_v9i8e25716_app4.docx]

**Appendix 4: A detailed description of the contents of the interventions included in the systematic review**

| **Title of intervention and authors employing intervention [in-text citation]** | **Description and contents of the intervention** |
| --- | --- |
| SPARX [49, 50, 51] | A computerised CBT programme consisting of 7 modules in a game-world the user works through. The components of SPARX include CBT, psycho-education, relaxation skills, problem solving, activity scheduling, challenging and replacing negative thinking and social skills. An avatar guide talks about depression, mood and carries out safety checks and signposts the user to further help if they need it. At the start of each level the guide outlines the purpose of that level. The user inhabits a personalised character who must restore balance in the game world, using the skills that they develop against depression to do so. At the end of each level the guide enables the person to reflect on the skills from that level and how that can be applied to real life alongside homework challenges. |
| The Journey [52] | The Journey is a fantasy game delivered using a CD-ROM where the user selects an avatar to complete a quest through magical lands. There are 7 magical lands with different topics based on CBT. Prior to starting the topic a mood monitoring and recap of the previous topic are completed, along with a quiz to test that topic. Modules include agenda setting, interactive exercises, animations and videos and terminate with summaries and homework setting. The Journey is accompanied by a guidebook for the user to reflect on the module and to complete further challenges as part of the homework. |
| MoodGYM [53, 54] | MoodGYM is an online programme with 5 main modules to work through based on CBT. The components of MoodGYM include information, relaxation, problem-solving, dysfunctional thoughts, negative thinking, self-esteem, cognitive restructuring, assertiveness and coping with relationships. MoodGYM is self-paced which allows the user to return to the module at the point they finished previously. |
| Mobiletype [56] | Mobiletype (Mobile Tracking of Young People’s Experiences) is a self-monitoring program used as an initial pathway to care. Mobiletype asks young people to monitor their mood, stress and daily activities on a mobile application four times a day. This information is accessible to both the young person and their GP to help GPs better understand the young person’s mental health and to detect signs of depressive symptomatology and other mental health problems so that interventions can be implemented if needed. The activities that Mobiletype tracks include: current activities, location, companions, mood, recent stressful events, responses to stressful events, alcohol use, cannabis use, quality and quantity of sleep, quantity and type of exercise and diet. |
| Blogging [59] | Short for weblog, a blog is often written and saved onto a website and stored on a user’s personal web space area. A blog can be of any nature ranging from hobbies, to professional to personal interests. As an intervention, blogging was required to be completed twice a week, with participants spending at least 20 minutes per blog and writing at least 200 words for each blog. Blogs may be visible to the public or closed, where open blogs can be commented on by other people, acting as a social aspect to a blog. |
| Cognitive Bias Modification Training [60] | Cognitive Bias Modification Training consists of 20 sessions delivered through the internet and reminders that are delivered via email. The reasoning of these sessions are explained to participants before starting them. The components consist of 9 sessions of interpretive bias tasks and 8 sessions of attention bias tasks which both aims to modify negative interpretations and 3 further sessions to strengthen the association between social-evaluative situations and positive outcomes including word sorting and enhancing self-esteem. |
| Stressbusters [57, 58] | Stressbusters is a computerised CBT programme of 8 sessions including goal setting, getting activated, emotional recognition, noticing thoughts, thought challenging, problem solving, improving social skills, relapse prevention. Stressbusters uses interactive features to present the 8 sessions including videos, graphics, animations and printouts. Video inserts consist of teenagers who themselves are completing the programme, with voiceovers about doing so. Sessions must be completed linearly and the user’s compliance is measured through the completion of tasks such as homework, activity plans, quiz answers and mood ratings. |
| Shamiri-Digital [55] | Shamiri-Digital is an online intervention accessed through a web-browser which consists of three modules including growth mindset, gratitude and value affirmation. Growth teachers the learner about the brain’s ability to grow and respond to challenges, and the learner is asked to write their own story about a challenge they have faced and overcome. In the gratitude module, participants list three good things about their lives for which they are grateful. In the final module, value affirmation, the learner reflects on the importance of personal values, and are tasked with thinking about a time they have used their personal values to guide life decisions. The intervention can be accessed here: <https://thrive-online.shamiri.institute/> |
